# Application of Three-Dimensional Digital Technology in Orthodontics: The State of the Art

**DOI:** 10.3390/biomimetics7010023

**Published:** 2022-02-02

**Authors:** Inês Francisco, Madalena Prata Ribeiro, Filipa Marques, Raquel Travassos, Catarina Nunes, Flávia Pereira, Francisco Caramelo, Anabela Baptista Paula, Francisco Vale

**Affiliations:** 1 Institute of Orthodontics, Faculty of Medicine, University of Coimbra, 3000-075 Coimbra, Portugal; ines70.francisco@gmail.com (I.F.); madalenaprata@hotmail.com (M.P.R.); filipa.p.s.marques@gmail.com (F.M.); raqueltravassos.91@gmail.com (R.T.); mcal9497@hotmail.com (C.N.); fppereira_@hotmail.com (F.P.); anabelabppaula@sapo.pt (A.B.P.); 2 Coimbra Institute for Clinical and Biomedical Research (iCBR), Area of Environment Genetics and Oncobiology (CIMAGO), Faculty of Medicine, University of Coimbra, 3000-075 Coimbra, Portugal; fcaramelo@fmed.uc.pt; 3 Laboratory of Biostatistics and Medical Informatics (LBIM), Faculty of Medicine, University of Coimbra, 3004-531 Coimbra, Portugal; 4 Centre for Innovative Biomedicine and Biotechnology (CIBB), University of Coimbra, 3000-075 Coimbra, Portugal; 5 Clinical Academic Center of Coimbra (CACC), 3030-370 Coimbra, Portugal; 6 Institute of Integrated Clinical Practice, Faculty of Medicine, University of Coimbra, 3000-075 Coimbra, Portugal

**Keywords:** orthodontics, imaging, three-dimensional, virtual plaster models, virtual planning

## Abstract

Three-dimensional technologies are one of the most recent and relevant advancements in the field of Dentistry. These systems, including intraoral scans, 3D imaging exams (CAT scan, CBCT and MRI), CAD/CAM 3D printing devices and 3D computer software, have enabled clinicians to greatly improve patient care along with reducing treatment planning time. The present descriptive study aims to explore possible applications of 3D technologies during the diagnosis, treatment plan, case monitoring and result assessment in orthodontics. The overall upgrade provided by these technologies can improve the clinicians’ workflow and effectiveness by simplifying conventional techniques considered to be especially arduous.

## 1. Introduction

Three-dimensional technologies have been widely used in different areas of dentistry including orthodontics. In both orthodontics and maxillofacial surgery, the implementation of these systems has gradually changed the way clinicians perform their diagnosis, treatment plans, case monitoring and result assessment. These technologies replicate anatomical structures in order to present tridimensional anatomy with accuracy [1,2].

Facial soft and hard tissue assessment along with dental evaluation is essential to provide a proper diagnosis and treatment plan. Orthodontists commonly resort to bidimensional imaging techniques in order to register the craniofacial anatomy. However, these methods present some drawbacks in detection of facial asymmetries, retained teeth and distortion of cephalometric points outside the medial sagittal plane. The propagation of 3D technologies prompted the development of tools that allow the user to interpret 3D data and, therefore, research more complex cases on a craniofacial level. 

Despite their usefulness, these techniques are far from being routinely used. A study conducted in the United States concluded that, from 2002 to 2008, diagnosis and treatment plans with digital models only increased 11.4%, from 6.6% to 18% [3]. This situation can be attributed to factors, including the lack of knowledge about these technologies by clinicians, the learning curve required to use these systems, the difficulties in qualitative assessment associated with software development and the dose of radiation that is necessary to obtain a complete facial image [4,5,6]

The most popular 3D techniques in orthodontics are CBCT (cone beam computerized tomography), sterophotogrametry or 3D surface imaging systems, the digitization of 3D intraoral scans and 3D printing devices with CAD/CAM (computer-aided design and manufacturing) technology. These printers can be used to manufacture indirect bonding trays, surgical guides, orthodontic braces and clear aligners [1,7]. These methods are better suited than 2D records or solid records since they are easier to physically store, they are prone to less handwriting errors, and they provide a 3D evaluation of craniofacial structures [8,9].

The aim of this narrative review is to explore possible applications of 3D technologies during the diagnosis, treatment plan, case monitoring and result assessment in orthodontics.

## 2. Diagnosis

### 2.1. 3D Cephalometry

#### 2.1.1. CBCT Assessment

The cephalometric analysis is an elementary tool of orthodontics as it helps determine dental and skeletal relations in the craniofacial complex. Although this is widely accepted, geometrical distortions, craniofacial asymmetries, structure overlay and incorrect positioning of the head can all hinder two-dimensional assessment accuracy.

In the past, CAT scans were a staple for diagnosis in dental practices. However, concerns regarding the patients’ exposure to high levels of radiation quickly emerged, and, in 1996, the CBCT was created as an alternative with lower radiation levels. Despite the lower levels of radiation (when compared to a CAT scan) the levels remain significantly higher than with a lateral cephalogram, and this information should not be overlooked. Additionally, the current scientific literature does not state if 3D cephalograms can be used on every orthodontic patient. To order a CBCT, the European Commissions’ directives regarding CBCTs should be applied to both dental and maxillofacial radiology. More recently, the European Academy of Dental and Maxillofacial Radiology disclosed new criteria for the use of CBCT specifically directed at dentists and orthodontists [10].

Three-dimensional cephalometry, which is done using a CBCT exam, allows for a more detailed evaluation of the craniofacial structure. With this method, the clinician can more easily detect and quantify craniofacial asymmetries, longitudinal growth and subtle occlusal changes [11,12].

This exam is also extremely important in dental impaction cases. Whether the treatment is surgical and/or orthodontic, accurately locating the tooth position is of paramount importance. The information provided by the CBCT grants a more perceptible visualization of the impacted tooth and the adjacent structures. This contrasts with the two-dimensional imaging method where several images of the impacted tooth from different perspectives are required, making this a more unreliable method with lower resolution imaging [13].

**Figure 1:**
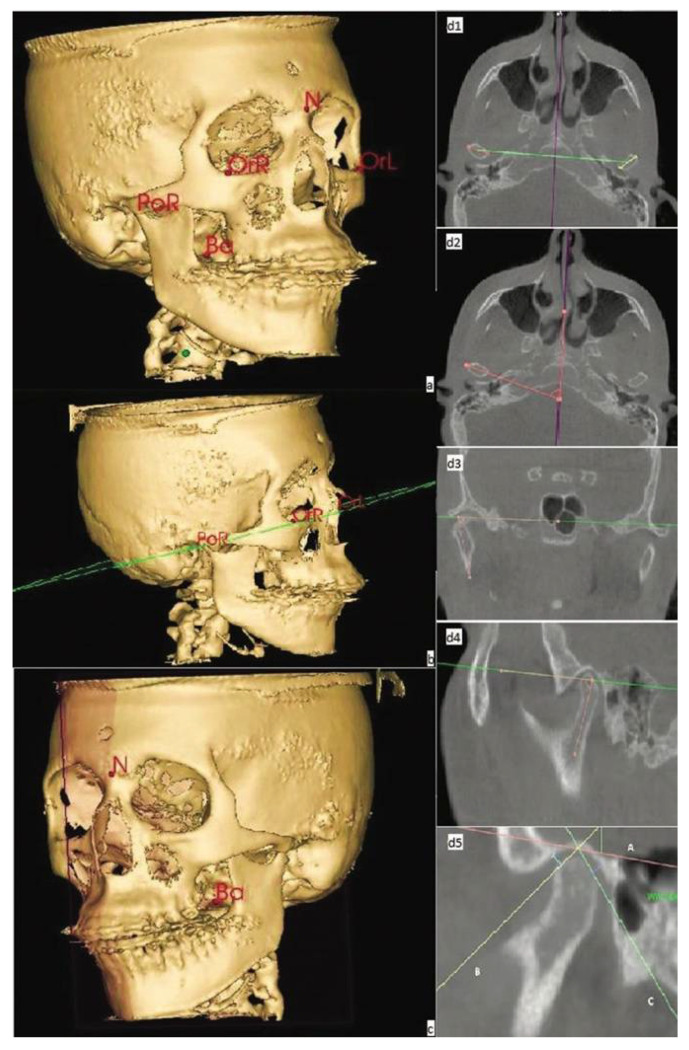
Three-dimensional cephalometry: Mean reference points (**a**); Frankfurt Horizontal Plane (**b**). Midsagittal plane (**c**). Intercondylar distance assessment (**d1**). Condyle angle (**d2**,**d3**) and Condyle position in the sagittal plane (**d4**,**d5**). N–nasion; OrR–Orbital right; OrL–Orbital left; PoR–porion; Ba–basion.

Recent studies have shown that 3D cephalometric images are not only accurate but also comparable to direct measurements of the cranium and conventional cephalometric analysis [14]. However, more studies are still required to introduce new points and planes. Some of the references that are used in 2D assessments cannot be applied in 3D evaluations since these will change their spatial expression. For example, a line on a 2D image represents a plane on a 3D image (Figure 1) [15].

Despite the known advantages of this method, further studies must be conducted in this area, regarding not only CBCT but also magnetic resonance imaging [11] since these appear to be highly compatible with CBCT-based cephalometry in both angular and linear measurements while not exposing the patient to radiation [14].

#### 2.1.2. Airways Measuring Method with CBCT

**Figure 2:**
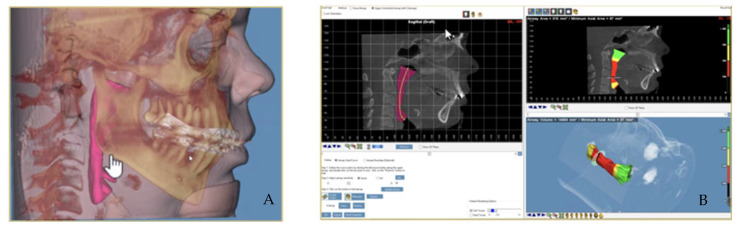
Airway measurement with CBCT: anatomic boundaries of the upper pharyngeal airway (pink) (**A**). Segmentation of the upper airways: nasopharynx (green), oropharynx (red) and hypopharynx (lower yellow) (**B**).

Airway three-dimensional measurements can be accurately obtained with the use of a CBCT. This has been a useful tool for patients with obstructive sleep apnea syndrome (OSAS) allowing for a comparison of airway constriction before and after undergoing different types of treatment plans (Figure 2).

The pharyngeal area can be described and subsequently measured by using different points and planes. According to Cossellu et al. this area can be divided into three planes: the superior plane (which goes from the basion to the posterior nasal spine), the medial plane (that is parallel to the cranial base plane and goes through the medial point of the first vertebra) and the inferior plane (which goes through the antero-inferior point of the first vertebra to the ment) [16]. However, Schendel et al. proposed a different assessment with the combination of the retropalatal space (extending from the posterior nasal spine to the inferior edge of the soft palate) and the retroglossal space (from the inferior edge of the soft palate to the hyoid bone) [17].

Brunetto et al. suggested an airways volume measurement with the combination of two sectors. The upper sector, VolA, is determined using planes that are either parallel or perpendicular to the FHP and anatomical limits, such as the sphenoidal sinus and cervical vertebrae. VolB is the lower volume sector and its anterior and posterior limits are the same as with VolA; however, the upper limit coincides with the VolA lower limit, and its lower limit is traced using a line parallel to the FHP that goes through the antero-posterior point of the fourth vertebra [18].

The nasal cavity can also suffer changes in situations of maxillary expansion. When attempting to assess these changes, linear measurements are taken from the first premolar, the first molar and the nasal base width. In the case of the first premolar, the distance is measured between cuspids or between root apex. In the evaluation of the first molars, the distance is measured between the disto-vestibular cuspids or the apexes of the vestibular roots. The nasal base width can be assessed by measuring the most far left and most far right points of the intersection between the maxillary sinus and the nasal cavity [19].

These methods have been proven effective in airways analysis and are an important tool in accurate assessment of treatment success.

#### 2.1.3. 3D Imaging and Spheno-Occipital Synchondrosis Ossification

The spheno-occipital synchondrosis is a cartilage that merges the sphenoid bones’ body with the basilar portion of the occipital bone, and this is the last cartilage to fuse in the body. The growth of this structure will influence the antero-posterior dimension of the cranium, and an improper fusion can lead to the development of malocclusion.

**Figure 3:**
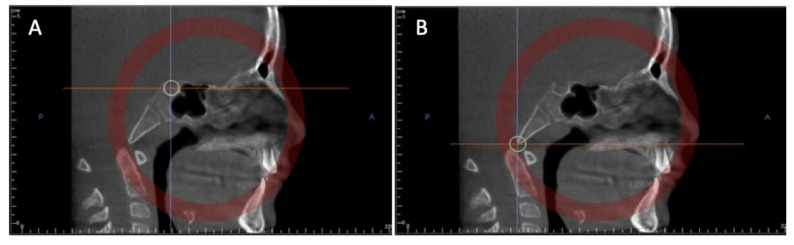
Spheno-occipital synchondrosis ossification assessment: Middle of sella turcica in the sagittal view (**A**). Anterior border of the foramen magnum in the sagittal view (**B**).

Through CBCT imaging, it is possible to assess the ossification stage of synchondrosis (Figure 3). The method used to determine the midsagittal plane for this assessment is composed of several planes. The first two planes go through the middle of sella turcica both in the sagittal and axial planes, and the other two planes intersect with the anterior border of the foramen magnum with one in the sagittal plane and the other in the axial plane. This can help predict possible growth patterns and subsequent treatment plans that might minimize any undesirable outcomes [20].

### 2.2. Treatment Diagnosis Using a 3D Scanner

The first orthodontic digitation system was developed by Cadent in 1999, and this technology has greatly improved in recent years [1]. With this technology, the clinician can create a three-dimensional image of the dental arches, either independently or in occlusion, from plaster models or impressions or by directly scanning the oral cavity [1]. The digital study models offer certain advantages when compared to their traditional counterparts, including better tolerance, more comfort, lower risk of allergic reactions as well as easy storage, recovery and sharing of data between peers [1,21]. The time it takes to scan the dental arches is variable and dependent on operator experience and technique [1].

Some studies that assessed diagnosis precision and measurement sensitivity on orthodontic digital models and plaster models have been conducted in the past [14,21]. A recent study revealed that there were no statistically significant differences when comparing measurement of the mesio-distal diameter of teeth between 3D models and traditional models measured with a caliper rule. Furthermore, the systematic review done by Gabrielle Rossini et al. showed that digital models are not only highly precise and reliable but also easy to reproduce [22].

## 3. Treatment and Monitoring of Orthodontic Treatment

### 3.1. 3D Imaging for Indirect-Direct Bonding

The key to a correct orthodontic movement is proper bracket placement. A precise bonding of the bracket will avoid the need for posterior re-positioning or bends on the archwire later on. The development of positioners has greatly improved the direct bonding of brackets; however, this process is still subject to human error. Indirect bracket bonding techniques have been developed in order to diminish human errors related to anatomical and morphological variations of crowns.

The evolution of imagological techniques, such as the CBCT, have improved diagnosis and treatment due to its three-dimensional vision of the patients’ soft and hard tissue. In order to better visualize both crowns and roots during bracket bonding, 3D images of the appliance kit and the patient that will be subjected to treatment are obtained. The roots are then isolated in the software. Afterwards, the brackets are placed on the crowns and meticulously adjusted. 

An image with a “U” molding tray is then added to the projection and subsequently adapted to the teeth and brackets. This molding tray will be covering the occlusal half of the teeth and brackets while leaving the other half uncovered. The images of brackets and teeth are then removed, and we are left with a bonding guide ready for printing. After printing and adjusting the tray, an indirect bonding can take place minimizing human error [23].

### 3.2. Clear Aligner Manufacturing

Clear aligners are based on systems that are highly reliant on 3D technologies, such as CBCT and especially intraoral scanners. With the use of the intraoral scanner, the orthodontist can create 3D models of the patients’ mouth and set up the orthodontic movements desired with the use of a specific automatic adjustment software for the soft tissues.

In a singular clear aligner case, several trays are needed in order to achieve the projected result. The aligner sequence is responsible for a gradual orthodontic movement and as such, for each movement, there is a different model and subsequently a different tray. After diagnosis, the orthodontist must define the intended outcome on the software prior to treatment. During this stage, the clinician must also divide the desired dental movements, which will result in the number of aligners necessary for the projected results.

After all the projections, each 3D model is converted into an STL file so that they can be 3D printed. These models will then be used to make the aligners with thermoformed plastic [24].

### 3.3. Surgical Guide Technique for Miniscrew Placement

The use of miniscrews in orthodontics has notably increased in recent years. Miniscrews allow for the simplification of several treatment plans by producing skeletal anchorage. Since these are easy to place and remove and require no cooperation from the patient, there has been an increased use of these devices in orthodontic practices. However, the stability heavily relies on bone thickness both on a vestibular and palatal level. Miniscrews are also difficult to correctly place, and there is a risk of dental damage, sinus or nasal perforation, chronical sinus inflammation and even anchorage loss [25].

Bone thickness varies from individual to individual so orthodontists must always assess each case before placing any miniscrew. The development of three-dimensional imaging exams has improved diagnosis and treatment plans by eliminating issues related to two-dimensional imaging [26].

**Figure 4:**
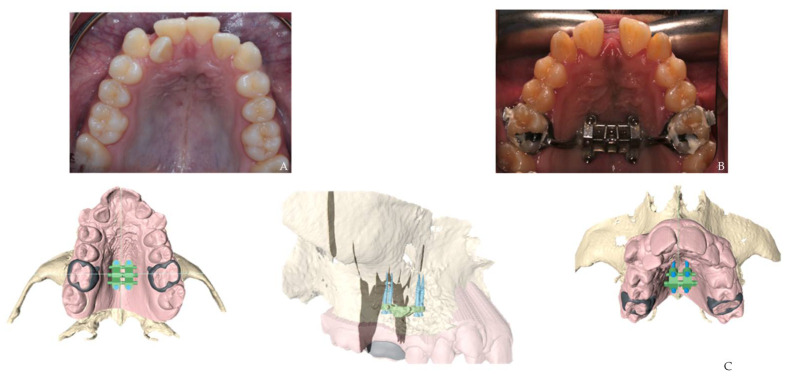
Planning the placement of micro-implants in the maxillary expander: Initial occlusal photo (**A**). Placement of the microimplant assisted rapid palatal expander (**B**). Microimplant placement planning through the superimposition of CBCT and digital models (**C**).

Through the superimposition of CBCT and digital models (intraoral scan), it is possible to evaluate the best placement area for miniscrews (Figure 4). After determining the preferred placement site, a surgical guide can be manufactured using a 3D printer. These guides will provide a precise and controlled placement while simultaneously minimizing risks commonly associated with this procedure [25,26,27].

### 3.4. Corticotomy Technique Using CAD/CAM 3D Printed Surgical Guides

A corticotomy is defined as a premeditated defect that is inflicted on the cortical bone, decreasing its resistance and drastically reducing treatment time. This technique is considered to be the only effective and low risk intervention that accelerates tooth movement.

This approach is commonly referred to as a corticotomy-assisted orthodontic treatment (CAOT), and it consists of administering small cuts along the area of alveolar bone where the movement are desired to take place. Although this method does present many advantages, corticotomies are, many times, still responsible for significant post-surgical discomfort. In order to bypass this possible discomfort, a minimally invasive procedure has been developed. This intervention relies on 3D-printed CAD-CAM surgical guides that have piezo surgical micro incision lines.

In order to manufacture these guides, first, a preliminary impression of both arches extending to the vestibule is taken, and an individual tray is manufactured in order to make a second impression that will be subsequently digitized with a 3D scanner. The model images and matching acrylic surgical template are stored as an STL file. The patient will then do a CBCT from which DICOM files can be extracted and subsequently combined with the use of a 3D image segmentation software that will transform these images into 3D models and save these models as STL files.

Afterwards, both arch STLs, scanned impressions and the acrylic surgical template will be combined to more easily visualize the 3D model in different perspectives and planes. The space between dental roots is assessed, and a longitudinal axis is traced along each tooth. Taking into account the direction of this pre-determined axis, a parallelepiped is projected and then removed from the 3D model leaving gaps for the piezoelectrical cuts. These gaps are projected to be 2 mm from the papilla all the way to the lower limit of the vestibule and 2 mm from the radicular apex.

The acrylic templates’ 3D model is transformed into a surgical guide with grooves projected for the scalpel blade first and then the piezoelectrical cut insert. Lastly, the surgical guides’ STL 3D model is printed [26].

### 3.5. Assessment of Tooth Movement in a Three-Dimensional Plane

The magnitude of orthodontic movement is reliant on several factors, such as the amount, duration and direction of the applied force; the force-momentum ratio; and the supporting periodontal tissues.

Dental movement assessment is commonly achieved by resorting to a panoramic radiograph, which should be done before, during and after treatment. However, this exam is not accurate for root position assessment since it suffers distortions. CAT scans and CBCTs are far more accurate and precise for this purpose. However, since orthodontic treatment requires frequent root movement monitoring, resorting to a CBCT or a CAT scan every single time would expose the patient to increased levels of radiation. In a recent study, a new methodology suggested that by combining the CBCT with intraoral scans and the digital models obtained with it, the clinician would be able to assess the root positioning at any time without exposing the patient to more radiation [28].

The use of digital orthodontic models is convenient for the orthodontist since the transfer, recovery and storage of data is relatively easy. These models are simple to manage for assessment of the mesiodistal crown diameter, arch length and dental crowding allowing for a smoother diagnosis process especially in extraction cases.

**Figure 5:**
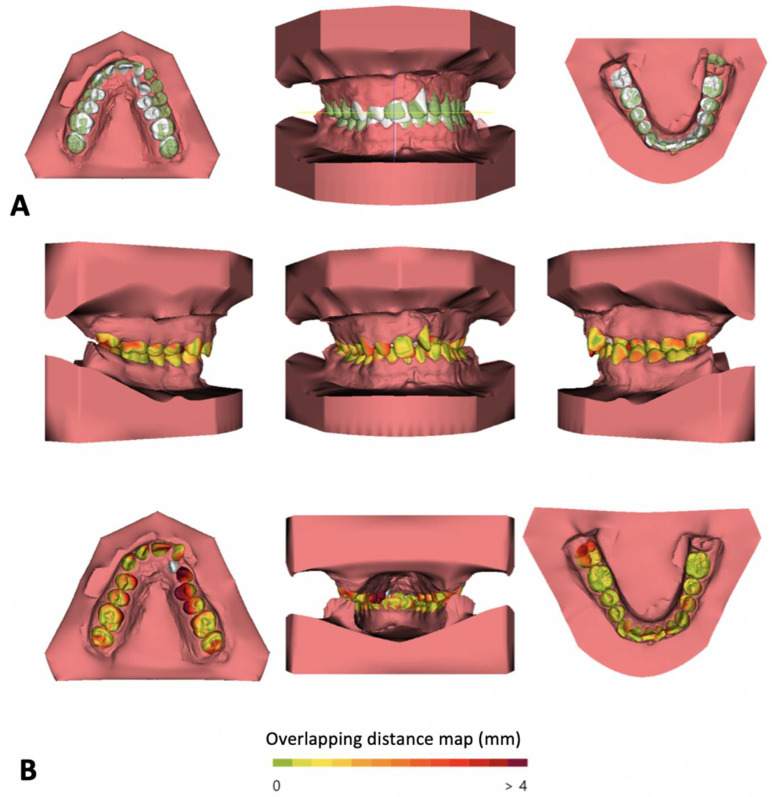
Three-dimensional tooth movement evaluation: Overlapping of the models before and after maxillary expansion- contacts after expansion represented in green color (**A**). Superimposition of models using a distance map (**B**).

In order to adequately appraise dental movement, impressions are taken before and after treatment. The resulting models are subsequently digitized using a 3D scanner and exported as STL files. Posteriorly, with the use of a suitable software program, the models are overlapped using two reference points: the distal extremity of the incisal papilla and a point distal to the first point of the medial palatine raphe. After this initial superimposition, the software will automatically project a 3D reference plane on the palatal region and this will serve as a guide to accurately overlap the models (Figure 5).

The current literature demonstrates that this method is more reliable when compared to its traditional counterpart. It is worth mentioning that, although this method is more dependable, it cannot be applied on all situations, for instance in the case of growing patients and patients subjected to orthopaedic or orthodontic-surgical treatments [29,30].

### 3.6. Distraction Osteogenesis

When distraction osteogenesis was introduced in 1992 [31], it revolutionized orthognathic surgery especially in cases of extensive bone lengthening with no possibility to resource to grafting. The distraction process is based on a gradual and progressive lengthening in which a bone callus will form in the place that suffered osteotomy.

The two types of distractor (intraoral and extra-oral) offer different sets advantages and disadvantages. Extra-oral distractors are attached on the patients’ face and connected to the bones with screws; this type of distractor is extremely advantageous since it allows the surgeon to have maximum control over the vector used for bone movement and adequately adapt it to the circumstances of specific cases. On the other hand, these devices are visibly striking and are used, on average, for a 7-month period, which is not easily accepted by many patients. 

Intraoral distractors have emerged as a response to the aesthetic concerns and discomfort caused by extra-oral distractors increasing patient approval. Initially, these appliances had a single movement vector that could not be changed post-surgery. The direction of the movement was solely dependent on placement during the surgery, this meant that, during the intervention, the surgeon had to confirm the distractor’s position in order to avoid an undesirable outcome. 

Furthermore, intraoral distractors are harder to correctly place since the surgical field is limited to the oral cavity. In children with anatomical deformities, such as Treacher–Collins Syndrome or Pierre Robin Sequence, this becomes an even more complex challenge due to their limited opening of the mouth. This creates various difficulties for the surgeon since, in order to not change the proposed vector of distraction, the device must be placed correctly along the patients’ jaw.

**Figure 6:**
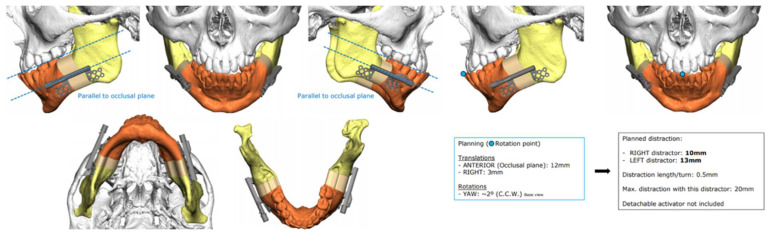
3D planning of intraoral distractor position with postoperative virtual simulation of the predicted results on hard tissues after repositioning the mobilized bone structures.

Computer-assisted surgical planning gives maxillofacial surgeons various tools to more accurately predict and assess different surgical approaches and possible outcomes (Figure 6). The three-dimensional planning makes it possible to test the distractor size and its performance with different vectors of distraction. These systems can simultaneously be used to plan surgical cuts making it possible to avoid any important anatomical structures, such as the inferior alveolar nerve or developing teeth. These types of planning systems have simplified a previously arduous task whilst also maximizing the treatment possibilities and improving the final outcomes [31].

### 3.7. Orthognathic Surgery

#### 3.7.1. Photorealistic Visualization of Rendered CT Images

The CBCT exam is necessary to accurately visualize soft tissues. However, these do not have a photorealistic appearance, which is imperative in cleft lip and palate diagnosis, scarring assessment and orthognathic surgery prognosis.

**Figure 7:**
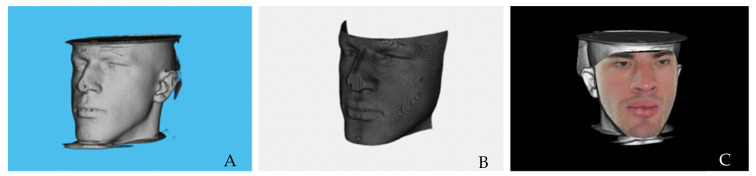
Combination of stereophotogrammetry images with CBCT images to create 3D virtual models of the patient: untextured soft tissue surface of the CBCT scan (**A**,**B**). Fusion of a CBCT soft tissue image with 3D stereophotographic images (**C**).

As an alternative, the photorealistic virtual face is a method that involves the overlaying and fusion of a CBCT soft tissue image with 3D stereophotographic images of the patient [32,33]. Stereophotogrammetry has evolved in recent years, and these methods now require two images one of each side of the patients’ face to create a 3D model that can be observed and measured regardless of perspective (Figure 7) [34]. Computer technologies have improved these techniques making the capture and building systems faster, simpler and more precise [6].

Studies have validated the accuracy of the superimposition of stereophotogrammetry images with CBCT images to create 3D virtual models of the patient for diagnosis and development of craniomaxillofacial treatment plans. According to Ayoub et al., the surface register minimal error is 1.5 mm [35].

#### 3.7.2. Orthognathic Surgery 3D Planning—Surgical Splint Manufacturing

Conventional orthognathic surgery planning relies on computer assisted two-dimensional surgical simulation systems, which integrate photographs and cephalograms as a guide [36,37]. Along with these systems, a facial arch is used in order to adequately register the patients’ bite in a semi-adjustable articulator. This process allows for a simulation of surgical movements using the patients’ cast models, which is necessary for surgical splint manufacturing [38]. Although this method is well established and widely used, it has a few setbacks, since the use of a conventional articulator and planning of three-dimensional procedures using two-dimensional imaging can create imprecisions [36].

Three-dimensional simulation systems with CBCT have arisen as a solution to some of the issues previously mentioned with two-dimensional planning [36,37,38]. These techniques require a CBCT, and, to perform this exam, the patient must have the head and muscles in a natural position with a relaxed expression while biting in centric relation [36,39]. Skin texture and structures must be refined and improved in order to accurately overlay with the CBCT reconstruction, and this can be done by mapping 2D photographs, using 3D photographs or a 3D surface scan with CBCT reconstruction data. 

When it comes to intraoral structures and dental artefacts, this refinement can be achieved through the digitization of the plaster casts, direct intraoral 3D scanning and scanning of the dental impression with a CBCT or a surface laser scanner [36,37,38]. The scan should be done immediately after the CBCT in order to avoid any issues during the rendering process. The bite register must be obtained with precision, and, in cases of functional deviations or double bite with multiple interferences, several bite records should be done in order to avoid imprecisions [39]. 

**Figure 8:**
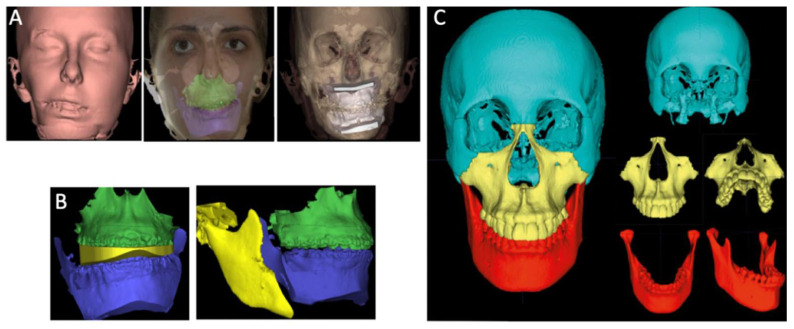
3D Orthognathic Surgery planning: Preoperative virtual simulation (**A**). Predicted results on hard tissues after repositioning the mobilized bone structures: intermediate virtual splint and final occlusion (**B**). Postoperative virtual simulation of the predicted results on hard tissues after repositioning the mobilized bone structures (**C**).

Using this approach, the segments are repositioned by translation movements in relation to the three spatial planes (*x*, *y* and *z*), and adjustments are made by rotation around these axis representing “roll, pitch and yaw” (Figure 8) [36,37]. Furthermore, it is possible to identify relevant surrounding structures that can interfere in the osteotomy process, such as the maxillary sinus, dental roots and inferior alveolar nerve [36]. After the 3D surgical planning, the surgical splints a can be manufactured using CAD/CAM techniques. Guide models to predict surgical cuts and the location of screws and surgical plaques can be planned in the 3D program [36,39].

When compared to its conventional counterpart, this technique appears to have a few advantages, namely, the possibility to detect some deformities or asymmetries that would have otherwise gone unnoticed, the capacity to simulate different surgical procedures, the identification of possible complications and the ability to correct the centric relation position on a temporomandibular level [40]. Another advantage of this method is that it allows the clinician to easily share information with other peers [38].

In the current literature, although some of these three-dimensional systems and protocols are mentioned, none of them are referred to as the gold-standard for image superimposition [41].

## 4. Retention and Outcome Assessment

### 4.1. Retainer Manufacturing Technique Using CAD-CAM Technology

The use of CAD-CAM to design and fabricate orthodontic appliances has been reported in the scientific literature. Removable appliances, brackets, lingual arches, occlusal splints, Herbst appliances and others have been developed for specific situations by resorting to this technology.

Different methods have been described with similar base stages. Initially, dental casts are digitized, and then, through the use of 3D software, the appliances are designed. The virtual appliance is produced with a subtractive or additive technology. In the subtractive method, an appliance is made by milling the desired design from a block of material. On the other hand, the additive method is achieved by layering material until the chosen design is completed. Stereolithography is the process of 3D fabrication by the additive method. This type of 3D printing is preferred to the subtractive technique in terms of the cost, speed, quality and capacity to manufacture complex structures [42].

Fixed retainers are placed lingually after finishing the orthodontic treatment and usually extend from canine to canine. These retainers can be 3D printed, and, by using this method, there is an improved placement accuracy [43].

### 4.2. Three-Dimensional Prognosis of Dental Arch Shape after Orthodontic Treatment

Orthodontic treatment plans should take into account the initial dental arch shape in order to achieve stable results. During the diagnosis stage, the arch shape should be evaluated (square, oval or conical), and the archwires used should be chosen according to their compatibility to each of these shapes. This technique relies solely on the operators’ perception leaving it susceptible to mistakes. As such, 3D software has been developed to help better choose adequate archwires for each individual case.

When compared to the conventional method, three-dimensional systems have proven to be more accurate in dental arch shape assessment and final prognosis. Furthermore, these systems can provide the orthodontist quantitative difference values between the dental arch and existing archwires. However, this method is not ironclad, as studies suggest that the final prognosis accuracy is only of 60% due to factors, such as biological individuality, orthodontic biomechanics and supporting tissues [44].

### 4.3. Superimposition on Outcome Analysis

As it was previously mentioned, cephalometric analysis is one of the pillars of orthodontics, and 3D exams, like the CBCT, have gained popularity in dental practices due to their accuracy and reliability. In three-dimensional cephalometry, the tracing and measurements are taken from actual structures; whereas, in two-dimensional cephalometry, these are done on projections of certain points, some of which are difficult to identify due to the overlay of other structures in the image. 

In the current literature, it is reported that the most reliable planes for superimposition are the maxillary plane (plane extending from the anterior to the posterior nasal spine) and the GO-ME plane. Therefore, the 3D technique is less prone to error, which ultimately leads to more precise cephalometric analysis [45].

## 5. Discussion

The topic of the study is recent and thus there are no studies with standardized methodologies in the current literature that allow synthesizing the studies in a systematic review with qualitative and/or quantitative analysis. This justified the choice of the authors to conduct a narrative review of the topic. Despite this, the realization of this type of study allows the summary of primary studies from which conclusions may be drawn into a holistic interpretation, permitting optimized workflow during diagnosis, treatment planning, case monitoring and outcome assessment. This review will inform clinicians of the latest 3D technologies in orthodontics, which will allow them to optimize the working time and improve the quality of treatment provided and, consequently, achieve improvements in patient care.

## 6. Conclusions

Three-dimensional technologies have evolved exponentially in recent years, and this has translated into great advancements in orthodontics. These advancements result in an optimized workflow during diagnosis, treatment planning, case monitoring and outcome assessment of any given case. A thorough study of these technologies will allow the orthodontist to optimize their time and knowledge and subsequently upgrade the quality of the provided treatment. These systems will continue to evolve to better suit the clinicians’ needs, and this will ultimately result in improved patient care.
